# Design and Implementation of Foot-Mounted Inertial Sensor Based Wearable Electronic Device for Game Play Application

**DOI:** 10.3390/s16101752

**Published:** 2016-10-21

**Authors:** Qifan Zhou, Hai Zhang, Zahra Lari, Zhenbo Liu, Naser El-Sheimy

**Affiliations:** 1 School of Automation Science and Electrical Engineering, Beihang University, Beijing 100191, China; zhanghai@buaa.edu.cn; 2 Geomatics Engineering Department, University of Calgary, Calgary, AB T2N 1N4, Canada; zlari@ucalgary.ca (Z.L.); zhenbo.liu2@ucalgary.ca (Z.L.); elsheimy@ucalgary.ca (N.E.-S.)

**Keywords:** wearable electronic device, foot moving direction, game play

## Abstract

Wearable electronic devices have experienced increasing development with the advances in the semiconductor industry and have received more attention during the last decades. This paper presents the development and implementation of a novel inertial sensor-based foot-mounted wearable electronic device for a brand new application: game playing. The main objective of the introduced system is to monitor and identify the human foot stepping direction in real time, and coordinate these motions to control the player operation in games. This proposed system extends the utilized field of currently available wearable devices and introduces a convenient and portable medium to perform exercise in a more compelling way in the near future. This paper provides an overview of the previously-developed system platforms, introduces the main idea behind this novel application, and describes the implemented human foot moving direction identification algorithm. Practical experiment results demonstrate that the proposed system is capable of recognizing five foot motions, jump, step left, step right, step forward, and step backward, and has achieved an over 97% accuracy performance for different users. The functionality of the system for real-time application has also been verified through the practical experiments.

## 1. Introduction

In recent years, with the rapid development of MEMS (Micro-Electro-Mechanical System) technology, the inertial sensor production has made a leap forward in terms of chip-size minimization, low-cost manufacturing, low-power consumption, and simplification in operation. Due to these advancements, various types of inertial MEMS sensors have been adapted for multiple applications, such as vehicles and personal navigation [1], motion tracking systems [2], and consumer electronic devices (smartphones) [3]. The wearable electronic devices, which emerged during the last few years, also utilize the low-cost MEMS inertial sensor, and are becoming more attractive in the consumer market. 

Wearable electronic devices refer to electronic technologies or devices that are incorporated into items of clothing and accessories which can be comfortably worn [4]. Generally, these devices can perform communications and allow the wearers to access their activity and behavior information. The foot-mounted inertial sensor based electronic device is one commonly existing type and has attracted attention for further study, development and implementation. The application fields of foot-mounted wearable device can mainly be categorized into: pedestrian navigation, human daily or sports activity recognition, and medical field.

The foot-mounted personal navigation system is the most universal usage of this kind of device and has been reported several times before [5,6,7]. It makes use of the self-contained and autonomous attributes of inertial sensor to derive the navigation solution, which is capable of effectively avoiding the negative effect of environmental elements. This system is meaningful to be utilized for firefighters, tracking military personnel, first responders and offenders, visually impaired and blind people [8]. The fundamental of such system is to apply Inertial Navigation System (INS) mechanization equation to calculate navigation parameters (i.e., position, velocity, and attitude), and combines the Zero velocity update (ZUPT) technology to mitigate accumulated error and estimate the sensor error [9,10]. In order to improve the positioning performance, several other technical algorithms or estimation approaches, such as particle filter [11], integration with RFID (Radio Frequency Identification) measurements [12], map matching [13], and improvement on hardware structure [14] are also employed in the foot-mounted navigation devices. However, due to the requirement of initial position and the INS accumulated error caused by integral computation [15], the foot-mounted navigation system is rarely capable of acquiring long-term stable solution and is limited for further industrial utilization. 

Additionally, the foot-mounted wearable device is applied in the field of sports exercises or daily activities tracking. With the inertial sensor attached on shoe, gait cycle, daily energy expenditure for activities (i.e., running, and walking) and sportive activities can be fed back to the users [16,17]. The activity recognition process basically segments data, extracts features and classifies human motions [18]. Wang designs a walking pattern classifier to determine the phases of a walking cycle: stance, push-off, swing, and heel-strike. Chen introduces employing Hidden Markov Model (HMM) to pre-process the inertial sensor data and classify common activities: standing, walking, going upstairs/downstairs, jogging and running, and other similar research work can be found in literatures [19,20]. In industry, miCoach [21] and Nike+ [22] are examples of foot-mounted commercial wearable products for monitoring sportive activities. These fitness products provide the user with information on speed, distance, and energy cost, and have achieved a tremendous popularity among users. However, they are limited to the provision of the users’ general motion information, such as discrimination of movement activity from rest, classification of activities (i.e., running, walking, and sleeping), and the quantization of general movement intensity (i.e., percentage of time spent moving, sleeping, and sitting) [23]. These systems are merely data recorders or monitors, and their performance does not have a direct impact on the user experience because the user cannot identify whether the motion identification result is accurate or not.

In medical field, since gait disturbances are very common factors in patients with Parkinson’s disease, several research works concern with Parkinson patients who suffer from walking abnormality, and aim to help them in aspects of diagnosing, monitoring, and rehabilitating. Filippo [24] proposes a system that is able to provide real-time computation of gait features, and feeds back to the user in the purpose of helping him/her execute the most effective gait pattern. Joonbum [25] makes use of pressure sensor to monitor patients’ gait by observing the ground reaction force (GRF) and the center of GRF to give the quantitative information of gait abnormality. He [26] improves the work by integrating Inertial Measurement Unit (IMU), employing the HMM to identify gait phases and developing it into a tele-monitoring system. Moreover, Strohrmann [27] utilizes the motion data measured by inertial sensor for runner’s kinematic analysis to avoid risks provoked by fatigue or improper technique. Chung [28] compares the motion data of Alzheimer patient and healthy people collected during walking, and concludes that the Alzheimer patients exhibited a significantly shorter mean stride length and slower mean gait speed than those of the healthy controls. The foot-mounted device, which provides continuous physical monitoring in any environment, is beneficial in shortening patent’s hospital stay, improving both recovery and diagnosis reliability, and raising patients’ quality of life. 

This paper aims to introduce a novel application of foot-mounted wearable electronic device: game play. The inertial sensor has been successfully applied in game play scenario before and received massive attentions. The most popular and famous example is Wii remote controller, which integrates infra-red and three-axis accelerometer information to capture users’ hand motion and enables them to play games such as Golf, Tennis ball, Balling, etc. [29,30]. The MotionCore controller, proposed by Movea, can play the role of an air mouse and is employed to play Fruit Ninjia, or Shooting games. Shum [31] introduces a fast accelerometer based motion recognition approach and applies this technology to plays boxing game. Ernst [32] attempts an initial experiment of using wearable inertial sensor in game of material arts. These proposed or stated inertial sensor based game applications are all expressed in hand operating mode that users need to shake and swing their hands to interact with game operation in real time. To the best knowledge of the authors, attaching inertial sensor on shoe and using foot motion to play game has not been discussed in previous academic work or industry product; hence, it is a novelty of suggesting such game play manner. The main idea behind the proposed system is to identify human foot stepping directions in real-time and coordinate these directions to control the virtual player actions in game. In our work, one IMU configuration is selected to make it convenient and suitable for user to wear. The proposed motion identification procedure is then implemented in three successive steps: (1) the collected dynamic data are initially preprocessed to compensate sensor error (i.e., bias, scale factor error, and non-orthogonality error) and correct the inertial sensor misalignment during placement; (2) the peak points of acceleration norm are detected for acceleration data segmentation and the selected features (e.g., mean, variance, position change, etc.) in the vicinity of each peak point are extracted; and (3) the extracted features are finally fed into a machine learning process to train the classifier. Notably, to improve the robustness of the proposed system, each stepping motion type has its own corresponding classifier.

The advantages of proposed system can be described as follows: (1) it extends the current foot-mounted electronic wearable devices beyond pedestrian navigation or human activity recognition and monitoring to the game play field, (2) some of the kinetic games will not be limited to a confined space (i.e., living room) and specific game boxes (i.e., XBOX or Wii) anymore, and, on the contrary, will be playable almost anywhere or anytime on various terminals (i.e., smartphones or tablets); and (3) it introduces the possibility of building the low-cost, portable, real-time wearable exercising and entertainment platforms, with which people are able to conveniently perform virtual sports or exercise in an interesting manner without environmental constrains.

The paper is structured as follows: Section 2 introduces the main concept of the proposed system. Section 3 describes the system overview with the introduction of both hardware and software platforms. Section 4 illustrates the specific implementation of the foot motion detection algorithm. Section 5 shows the experimental results and analysis. Section 6 presents conclusions and provides recommendations for future research work.

## 2. Main Concept

**Figure 1:**
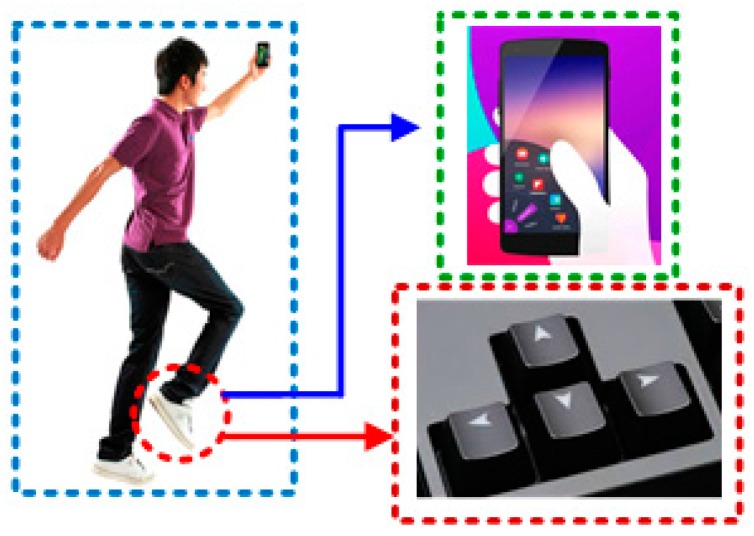
The main concept of proposed system.

One of the commonly-used game operating modes is that user controls the character’s movements (i.e., forward, backward, left or right) to avoid obstacles or achieve more points in popular smartphone running games, such as Temple Run, Surway Surf. According to this operation mode, the main concept of the foot-mounted systems is to utilize user’s steps to control the virtual player in game, instead of using the conventional manner (i.e., finger sliding, and button press). Specifically, the inertial sensor is attached to the user’s foot and the sensor data are collected during moving phase; then, the stepping direction is derived from collected data and used to control the character moving in game. Figure 1 illustrates the main concept of the proposed system.

This illustration shows the human foot’s kinetic motions, detected by an inertial sensor, will substitute the traditional game controller. Concretely, the user’s stepping forward or jumping correlates to the press of up button (or finger slides up); stepping backward correlates to the press of down button (or finger slides down). Similarly, a person’s walking left or right correlates to the press of left or right buttons (or finger slides right or left).

This system has high real-time and detection accuracy requirements because any lag or false detection of steps will cause the user to discontinue playing game normally and contribute to a poor user experience. Hence, the main challenge of this system is to correctly determine step motion and moving directions when the step event happens with little delay, and synchronize those motions to game controls, which is meaningful to provide a favorable feedback to user. Moreover, due to the diversity of shoe styles, sensor mounted manners and user habits, the system robustness and algorithm compatibility are other difficult challenges to overcome.

## 3. System Architecture

**Figure 2:**
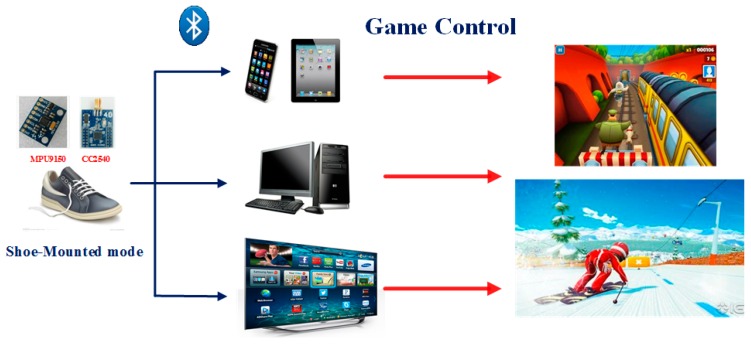
System architecture.

The proposed system architecture is shown in Figure 2. In this system, the foot moving dynamic data are captured by inertial sensor, and is then wirelessly transmitted to various kinds of terminals (i.e., smartphone, tablets, computer, and smart TV) through Bluetooth 4.0. The software, which is compatible in different platforms, plays the role of receiving data, performing the step motion detection algorithm, and interacting with games. Both hardware and software platforms are included in this system, and are described as follows.

### 3.1. Hardware Platform

**Figure 3:**
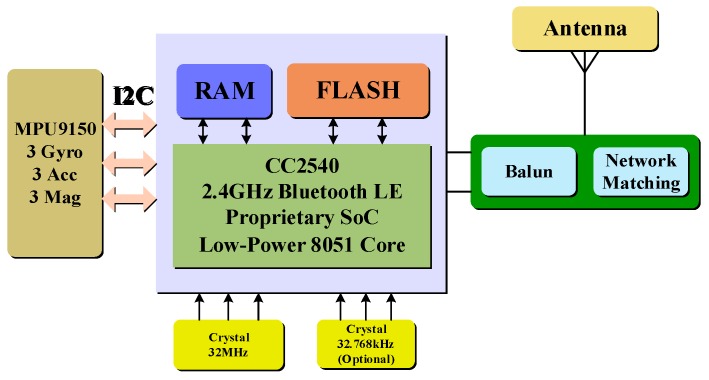
System hardware platform.

The system hardware platform mainly combines a CC 2450 microprocessor (Texas Instrument, Dallas, TX, USA), a MPU9150 9-axis inertial sensor (InvenSense, Sunnyvale, CA, USA), and other necessary electronic components. The CC2540 [33] processor has a high performance and low-power 8051 microcontroller and includes a 2.4 GHz Bluetooth low energy System on Chip (SOC). It can run both application and BLE (Bluetooth Low Energy) protocol stack, so it is compatible with multiple mobile devices (i.e., Smartphone, and tablets). The MPU9150 [34] is an integrated nine-axis MEMS motion tracking device that combines a three-axis gyroscope, a three-axis accelerometer, and a three-axis magnetometer. Figure 3 shows the system hardware platform. In our system, the three tasks of the hardware platform are to derive inertial sensor data through II2 (I2C) interface in a pre-set sampling frequency (200 Hz), to package data in a pre-defined user protocol and send data via Bluetooth to the host. 

### 3.2. Software Platform

The system software platform is developed in C++ programming language in Visual Studio. The main functions of the software include the following: receive and decode data, log user’s motion data, calculate the attitude, run the human foot detection algorithm and interact the human motion with game control. For real-time processing, a multi-threaded program is designed to simultaneously implement the listed tasks. Multithreading is a widespread programming and execution model that allows multiple threads to exist within the context of a single process. These threads share the processor's resources, but execute their functions independently. This multi-threaded software can guarantee the whole system’s real-time application, and moreover introduces a clear structure, which is beneficial for further revision or development.

## 4. Methodology

**Figure 4:**
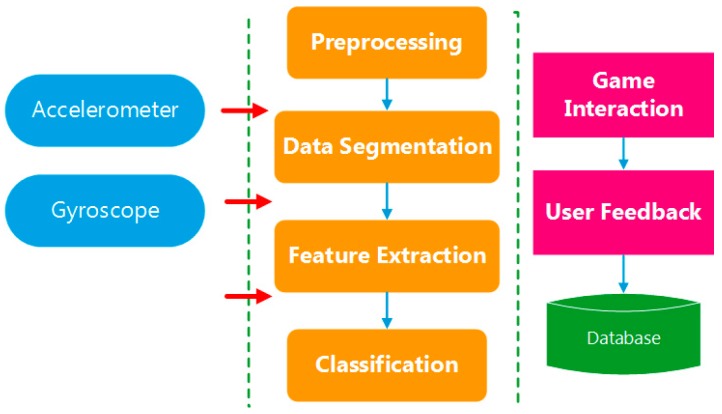
Overview of foot motion identification process.

The inertial sensor is attached on human foot and the measured rotation and acceleration information is applied for stepping direction classification. The motion recognition process of the proposed system is illustrated in Figure 4.

As shown in Figure 4, the identification process is executed as: first, the collected raw inertial data is pre-processed for error compensation, noise reduction and misalignment elimination; second, the peak points of the norm of 3-axis acceleration are detected to segment data; and, finally, the selected features in the divided data segment are extracted and put into the classifier to derive the foot motion types. The detailed description of each procedure is provided in the following subsections. 

### 4.1. Preprocessing

MEMS inertial sensor has the advantages of being small-size, low cost, affordable; however, they suffer from various error sources, which cause negative effects on their performance. Therefore, calibration experiments are indispensable to remove the deterministic errors, such as bias, scale factor, misalignment before using MEMS sensor. The inertial sensor error model [35] is described as follows and it is employed for the error compensation:

(1) $$\begin{array}{l} {\tilde{\omega }}^{b}=\omega +b_{\omega }+S_{\omega }\cdot \omega^{b}+N_{\omega }\cdot \omega^{b}+\varepsilon (\omega )\\ {\tilde{f}}^{b}=f+b_{f}+S_{f}\cdot f+N_{f}\cdot f+\varepsilon (f) \end{array}$$

where ${\tilde{f}}^{b},{\tilde{\omega }}^{b}$ denote the measurements of specific force and rotation and $f^{b},\omega^{b}$ denote the true specific force and angular velocity. $b_{a},b_{\omega }$, respectively, denote the accelerometer and gyroscope instrument bias; $S_{b},S_{\omega }$ separately denote the matrices of the linear scale factor error of gyroscope and accelerometer; and $N_{b},N_{\omega }$ denote the matrices of representing axes non-orthogonality. ε(ω),ε(f) denote the stochastic error of the sensors. The parameters $S_{b},S_{\omega },b_{a},b_{\omega },N_{b},N_{\omega }$ can be derived through a calibration experiment before sensor usage [36,37]. With a hand rotating calibration scheme, the experiment can be accomplished in approximately one minute [38]. 

In the proposed system, the IMU is attached on shoes to detect the user’s foot motions and control the game. However, due to the difference between various shoe styles and the sensor placement, the IMU orientation (pitch and roll) varies when mounting on different users’ shoes, which causes the misalignment with different users.

Hence, in order to achieve a satisfactory identification result for different shoe styles or placement manners, the data should be collected under various attachment conditions and put into the training process to derive the classifier. However, this process is time-consuming and the performance is not guaranteed if the sensor is attached with a new placement that is not included in the training set. 

**Figure 5:**
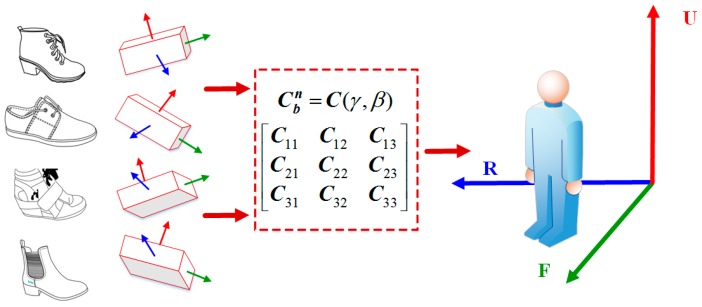
Inertial sensor measurement alignment.

To avoid such drawbacks, we propose to project the measured acceleration and rotation data from the sensor frame (shoe frame) to the user frame, where the user frame is defined as the user’s right, left and up directions as three axes to construct the right handed coordinate system. Thus, no matter how the inertial sensor is placed on the shoes (sensor frame is always different), the measured data can be unified to be expressed in the same coordinate. During the sensor installation, the forward axis of the IMU (*y*-axis in proposed system), is always aligned with the foot moving forward direction, so we only need to consider the misalignment of pitch and roll angles. This proposed data transformation from sensor frame to user frame is able to effectively eliminate the misalignment caused by different shoes styles and sensor placement because it aligns all the collected data in the same frame. Figure 5 shows this process.

Figure 5 shows the alignment process with the rotation matrix $C_{b}^{n}$, where the inertial data, collected under different misalignment conditions, are scaled in the same frame (Right-Forward-Up). More importantly, the data expressed in this frame can directly reflect the actual user moving direction in horizontal plane, which provides a better data basis for the consequent signal process, and is beneficial to achieve a more robust result. 

Therefore, a reliable and accurate attitude result is very significant and necessary since it can be used to correctly project the inertial measurement onto user frame with the rotation matrix to perform the data standardization process (align in the same frame), and consequently derive a dependable feature extraction section. Considering the given initial attitude and gyroscope measurement, the orientation results can be derived by integrating the angular velocity measured by 3-axis gyroscope. However, due to the error of MEMS gyroscope, the attitude result drifts quickly with time and is not able to provide long term solution. On the other side, the accelerometer can provide attitude angles without suffering from long term drift which is complementary with gyroscope, and is effective to compensate the attitude drift error. Hence, an attitude filter is used to integrate the gyroscope and accelerometer measurement together and derive the non-drift attitude solution. The Kalman filter is then used to blend the information in a feature-level fusion [39]. The dynamic model, measurement model of the filter and an adaptive measurement noise tuning strategy implemented are subsequently described as follows. 

#### 4.1.1. Dynamic Model

The attitude angle error model, which is the angle difference between true navigation frame and the computed navigation frame, is employed as the dynamic model [40]. This model is expressed in linear form, and easy to implement. The 3-axis gyro biases are also included in the dynamic model; and they are estimated in the filter and work in the feedback loop to mitigate the error from raw measurement. The equation of dynamic model is written as:

(2) $$\begin{array}{l} \dot{\psi }=\psi \times \omega_{in}^{n}+C_{b}^{n}\varepsilon^{b}\\ {\dot{\varepsilon }}^{b}=\left(-1/\tau_{b}\right){\dot{\varepsilon }}^{b}\;+\;\omega_{b} \end{array}$$

where ψ denotes the attitude error. $\omega_{in}^{n}$ denotes the *n*-frame rotation angular rate vector relative to the inertial frame (*i*-frame) expressed in the *n*-frame. $C_{b}^{n}$ denotes the Direction Cosine Matrix (DCM) from *b*-frame (i.e., the body frame) to *n*-frame (i.e., the navigation frame). The symbol “×” denotes cross product of two vectors. $\varepsilon^{b}$ denotes the gyros output error. Here, we only consider the effect of gyro bias and it is modeled as first order Gauss-Markov process. Finally, $\tau_{b}$ denotes the correlation time of the gyro biases and $\omega_{b}$ is the driving noise vector.

#### 4.1.2. Measurement Model

The acceleration residuals in the body frame are used to derive the system measurement model. In our model, instead of using attitude difference separately derived by accelerometer and gyroscope, the acceleration difference is applied to avoid the singularity problem when the pitch angle is ±90° [41]. The acceleration residuals in body frame are defined as the difference between accelerometer direct measurements and the projection of local gravity on the body frame.

(3) $$\begin{array}{l} \delta a=a_{m}^{b}-a_{n_{c}}^{b}\\ a_{n_{c}}^{b}=C_{n_{c}}^{b}a^{n} \end{array}$$

where $a_{m}^{b}$ denotes the accelerometer measurement. $a_{n_{c}}^{b}$ denotes the local gravity acceleration project on the body frame using the gyros derived rotation matrix $C_{n_{c}}^{b}$. The subscript $n_{c}$ denotes the computed frame. According to the DCM chain rule, $C_{n}^{b}$ is expressed as:

(4) $$\begin{array}{l} \;C_{n}^{b}=C_{n_{c}}^{b}C_{n}^{n_{c}}\\ C_{n}^{n_{c}}=I-[\psi \times ] \end{array}$$

where [ψ×] denotes the skew matrix of attitude error. Substituting Equation (4) into Equation (3), the relationship between acceleration residuals in body frame and attitude error is written as:

(5) $$\begin{array}{ll} \delta a & =a_{m}^{b}-a_{n_{c}}^{b}=C_{n}^{b}a^{n}-C_{n_{c}}^{b}a^{n}\\ & =\left(C_{n}^{b}-C_{n_{c}}^{b}\right)a^{n}=\left(C_{n_{c}}^{b}C_{n}^{n_{c}}-C_{n_{c}}^{b}\right)a^{n}\\ & =C_{n_{c}}^{b}\left(I-[\psi \times ]-I\right)a^{n}=C_{n_{c}}^{b}\left(-[\psi \times ]\right)a^{n}\\ & =C_{n_{c}}^{b}\left(\left[a^{n}\times \right]\right)\psi \end{array}$$

Then, the measurement model can be obtained by Equation (5). The measurement Z is the acceleration in body frame ${\left[\delta a_{x}\;\delta a_{y}\;\delta a_{z}\;\right]}^{T}$, and the measurement matrix H is expressed as:

(6) $$H=\left[\begin{array}{l} -gC_{n_{c}}^{b}(1,2)\;gC_{n_{c}}^{b}(1,1)\;0\;0\;0\;0\;\\ -gC_{n_{c}}^{b}(2,2)\;gC_{n_{c}}^{b}(2,1)\;0\;0\;0\;0\\ -gC_{n_{c}}^{b}(3,2)\;gC_{n_{c}}^{b}(3,1)\;0\;0\;0\;0 \end{array}\right]$$

This attitude filter works effectively under stationary or low acceleration conditions. In these situations, the specific force measured by accelerometer equals to local gravity acceleration, so the pitch and roll angles derived through accelerometer are accurate and can be positive in fixing the accumulated attitude error caused by gyroscope error, while, in high dynamic situation, the accelerometer will sense the external dynamic acceleration, which is undesirable in the filter. Hence, if the contribution of measurement update remains a same weight as that in low dynamic situation, a side effect will be introduced and lead to a degraded performance. Hence, to achieve an optimal attitude estimation result, we propose to adaptively tune the measurement covariance matrix R according to a system dynamic index ε [42] and is designed as:

(7) ε=|f−g|

where f denotes the norm of measured acceleration and g denotes the local gravity acceleration. Then the specific tuning strategy of covariance matrix R is described as follows:

1. Stationary mode: If the scalar subjects to ε<Thres1, the system is considered to be stationary. Correspondingly, the covariance matrix R is set as $R=diag[\sigma_{x}^{2}\;\sigma_{y}^{2}\;\sigma_{z}^{2}\;]$, where $\sigma_{x}^{2},\sigma_{y}^{2},\sigma_{z}^{2}$ denote the velocity random walk of three-axis accelerometer. In our approach, the Thres1 is set as $3\cdot (\sigma_{x}^{2}+\sigma_{y}^{2}+\sigma_{z}^{2})\;$.
2. Low acceleration mode: If the index satisfies the condition Thres1<ε<Thres2, the system suffers from low acceleration and is treated as measurement noise. The covariance matrix R is set as $R=diag[\sigma_{x}^{2}\;\sigma_{y}^{2}\;\sigma_{z}^{2}\;]+k\varepsilon^{2}$, where k is the scale. Thres2 is set as 2g
3. High dynamic mode: If the scalar subjects to ε>Thres2, norm of the three accelerations is far from the specific force, which equals to gravity acceleration. The acceleration residuals are not reliable. In this situation, we only use the angular velocity to calculate attitude, and the filter only performs the prediction loop without measurement update.

### 4.2. Data Segmentation

Data segmentation is carried out to divide the continuous stream of collected sensor data into multiple subsequences, and retrieve the important and useful information for the activity recognition. The sliding windows algorithms are commonly used to segment data in various applications because they are simple, intuitive and online algorithms. However, this approach is not suitable here because an entire human stepping motion signal may not be included in the current detected window, and is separated in two adjacent windows, which is possible to cause poor result in some cases. Moreover, this algorithm works with a complexity of O(nL), where L is the average length of a segment, and it affects the system real-time capability.

**Figure 6:**
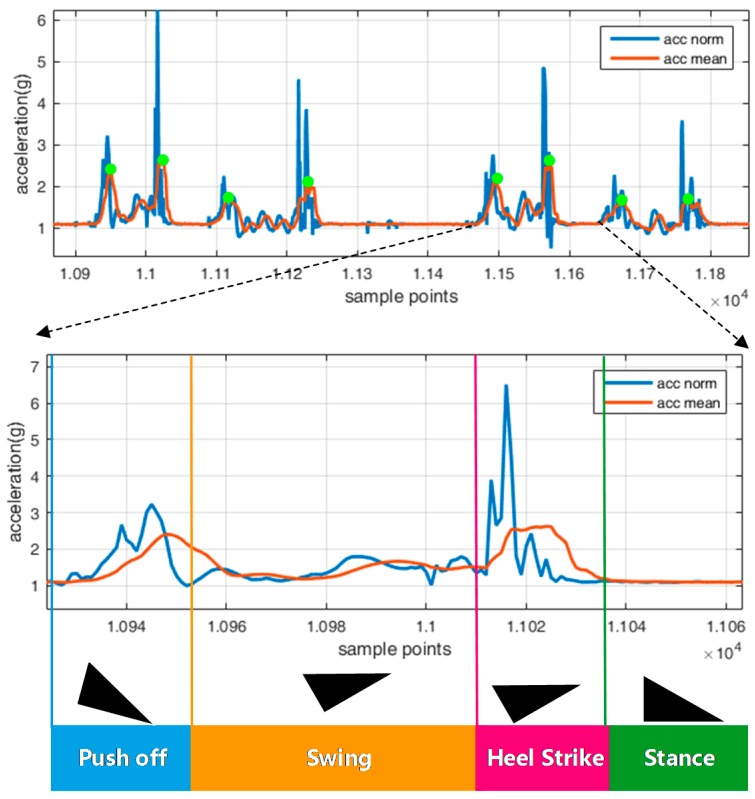
Stepping acceleration signal and gait phases.

Hence, the relationship between gait cycle and acceleration signal is analyzed to derive a practical approach to segment data. Generally, a gait cycle can be divided into four phases [43], namely: (1) Push-off, heel off the ground and toe on the ground; (2) Swing, both heel and toe off the ground; (3) Heel Strike, heel on the ground and toe off the ground; and (4) Foot stance phase, heel and toe on the ground at rest. Figure 6 shows these four phases and their correlated acceleration signal. 

As shown in Figure 6, the blue line is the norm of three accelerations and red line denotes the smoothed acceleration signal by a moving average algorithm, where, for each epoch, a window containing the previous N sample points is averaged to produce the acceleration value; and the reason is to derive a smoother form of signal, deduce noise, and eliminate unexpected peak points. 

Figure 6 illustrates that the smoothed acceleration signal during one walking cycle generally features two peak points, one is in the push-off phase that the foot is leaving the ground and another one is in the heel-strike phase that the foot hits the ground. Although it may not hold for each walking cycle, that more than two peak points are available in one cycle, due to different user habits of motion strength, these two points are always available in each gait cycle. Here, the utilization of the peak point for triggering the date segmentation process is proposed. Once one peak point is detected, the feature in the vicinity of this point is extracted and consequently the foot motion type is identified.

The reason for using peak point is that one peak point is always available in the push-off phase when the foot leaves ground, which will not vary for different users or stepping patterns. This point facilitates the detection of the beginning phase of each step, and ensures the reliable real time performance. On the other side, the foot motion detection algorithm works with the O (peak point number) complexity. Therefore, the classification process is only performed when the peak point is detected, which decreases the computation burden. Moreover, the specific phase of each walking circle do not need to be classified, as it simplifies the identification process 

Additionally, the length of data for feature extraction also needs to be ascertained. A tradeoff is available here between discrimination accuracy of motion types and real-time applicability. Involving more data in the segmentation procedure is beneficial to correctly identify human motion and achieve more reliable results, but will cause a lag response, whereas less data can achieve a quick and less delay judgment on human motion. However, there is not enough information included for classification. Hence, the distribution of three separate axis acceleration signal of different motions is analyzed to figure out the length of data segment for feature extraction.

**Figure 7:**
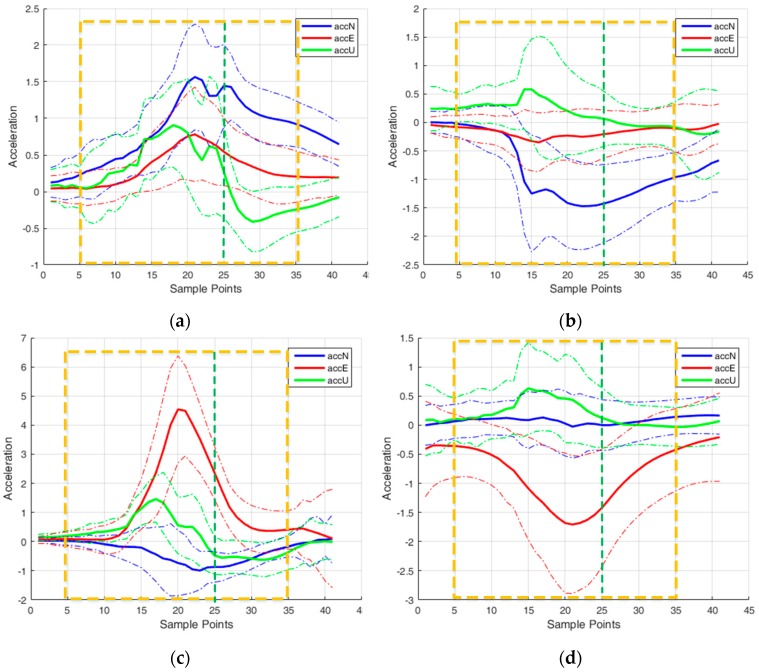

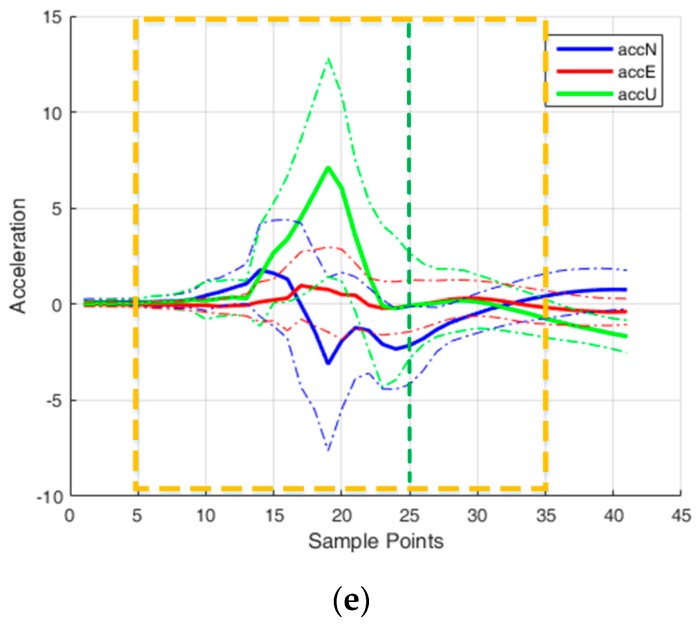
Acceleration signal of different motions and data segmentation: (**a**) Forward; (**b**) Backward; (**c**) Left; (**d**) Right; and (**e**) Jump.

Figure 7 draws the collected three axes acceleration signals in the vicinity of the peak points in the initial stage of a step, and Figure 7a–e, respectively, represents acceleration signals collected from forward, backward, left, right and jump motions. The blue, red and green solid lines separately denote the acceleration signals represented in user frame. The green dashed line, which drawn from top to bottom, denotes the position of the peak points. The peak points line suffers a shift in right side and it is due to the implementation of the mean average algorithm, but it will not cause any negative effect to the identification process. It is suggested to use the acceleration signals to invest the data segment length because they experience different performances during the process of human stepping in various directions, and they are able to provide an intuitive, direct, and easy understanding manner to recognize the moving directions. For example, Figure 7c illustrates the left motion and the acceleration (red line) in user’s right direction features an obvious difference compared with the other two axes. Similarly, for the forward and backward motions, the accelerations in forward or backward directions exhibit more diversity.

Additionally, each figure illustrates the acceleration distribution of 500 motion samples performed by different testers where for each motion 500 data groups are collected. Acceleration data in the vicinity of first peak point are extracted, and the mean and standard deviation of these segments are calculated. The solid lines and dashed lines represent the mean and standard deviation, respectively. The acceleration distribution shown in the Figure 7 provides an intuitive statistical result of acceleration in the initial phase of a step and is helpful to confirm the data segment length. The data segmentation length selected for feature extraction is 31 samples and it is presented in orange rectangle in the figure, where it includes 20 samples before the peak point with 10 samples after the peak point and the peak point itself. The main justifications to choose this length of data are: first, the extracted feature within the selected interval is able to provide enough distinguished information for the motion identification; and, second, it ensures a reliable real-time applicability. The data shown in Figure 7 is sampled in 200 Hz and the first 30 samples of a gait cycle are utilized for classification, which means that the motion type can be decided in approximately 0.15 s after this motion occurs.

### 4.3. Feature Extraction

Generally, features can be defined as the abstractions of raw data. The objective of feature extraction is to find the main characteristics of a data segment which can accurately represent the original data and identify valid, useful and understandable patterns. Basically, the features can be divided into various categories, time domain and frequency domain are the most commonly ones used for recognition. The feature selection is an extremely important step because a good feature space can lead to a clear and easy classification and poor feature space may be time-consuming, computationally-expensive, and cannot lead to good result. In our system, not all of the commonly-used features in activity recognition field in our system are selected; however, the collected signal is analyzed and the foot moving physical discipline is considered to choose the features, which are not only effective to discriminate motion types but also has less computation complexity. In this system, the selected features for foot motion classification are described as follows.

#### 4.3.1. Mean and Variance

The mean and variance value of the three axis accelerometer and gyroscope measurements are derived from the data segment to consider as the feature, according to the following equations:

(8) $$\left\{ \begin{array}{c} \bar{x}=\frac{\sum_{1}^{N}x_{i}}{N}\\ \sigma^{2}=\sum_{i=1}^{N}{\left(x_{i}-\bar{x}\right)}^{2} \end{array}\right.$$

where $x_{i}$ denotes the signal, *N* denotes the data length, and $\bar{x},\sigma^{2}$ denote the mean and variance value of the data sequence. 

#### 4.3.2. Signal Magnitude Area

The signal magnitude area (SMA or sma) is a statistical measure of the magnitude of a varying quantity, and actually is the absolute values of the signal. SMA is calculated according to Equation (9).

(9) $$f_{SMA}=\int_{t_{1}}^{t_{2}}\left|x\right|dt$$

where x denotes the signal and $\left(t_{1},t_{2}\right)$ denotes the integration time period. 

#### 4.3.3. Position Change

Position change is an intuitive feature for the foot direction identification because different foot moving directions cause various position changes. For example, jumping features a larger change in vertical direction, and stepping right and left lead to an obvious position change in horizontal plane. The Inertial Navigation System (INS) mechanization equation is able to provide the trajectory of a moving object in three dimensions with the measured rotations and accelerations [44], while, due to the double integration strategy of INS mechanization and the noise of sensor, the accumulated errors will be involved in the trajectory estimation and lead to a drift of position, especially when using a MEMS sensor. 

Hence, it is not feasible to calculate the position during the whole identification process, the position is only derived in the data segmentation, with an initial velocity (0,0,0), initial position (0,0,0), and a zero azimuth during the calculation process. The inertial sensor has the characteristic of keeping accurate in short term, so the position result computed in the 31 samples interval is reliable and trustworthy. The position calculation equation is described as follows:

(10) $$\left\{ \begin{array}{c} a^{n}=C_{b}^{n}a^{b}\\ v=v_{0}+\int a^{n}dt\\ p=p_{0}+\int v\;dt \end{array}\right.$$

where $a^{b}$ denotes the measured acceleration in body frame, $C_{b}^{n}$ is the rotation matrix that projects the acceleration from body frame to the navigation frame (local-level frame,) and $a^{n}$ denotes the projected acceleration in navigation frame. v,p denote the computed velocity and position and $v_{0},p_{0}$ denote the initial velocity and position.

#### 4.3.4. Ratio

The ratio feature is used to calculate the proportion of feature in single axis and the norm of features in three axes. The aim of introducing the ratio metric is to normalize the feature of three axes to best deal with the motions performed in different strength performed by different users. For example, for the jump motion, the position change in up direction (jump height) is more than that in horizontal plane and is dominant in the position change, though the jump height is different for various users, the proportion of jump height in position change still occupy significantly. Specifically, the position feature (the position change) derived from a heavy jump motion maybe (0.2, 0.2, 0.5), and is (0.05, 0.05, 0.2) from a slight jump motion; though the jump height amplitude varies significantly depending on different user habits, the ratio of jump height occupies over 50% of whole position change for the both groups. Hence, the ratio feature of position change in different directions is a good metric to distinguish and evaluate the motion types with different strengths. The ratio feature introduced here is calculated as in Equation (11):

(11) $$\begin{array}{c} \left\{ \begin{array}{c} FeatureNorm=\sqrt{FeatureX^{2}+FeatureY^{2}+FeatureZ^{2}}\\ ratioFeatureX=\frac{FeatureX}{FeatureNorm}\\ ratioFeatureY=\frac{FeatureY}{FeatureNorm}\\ ratioFeatureZ=\frac{FeatureZ}{FeatureNorm} \end{array}\right. \end{array}$$

where, FeatureX， Y ,Z denote the calculated features in different axes and ratioFeature denotes the ratio. In our proposed system, the position, mean, variance, and SMA features calculated in three directions or axes are all considered to derive the ratio feature. 

### 4.4. Classification

The classification is a process to predict or reorganize the motions with the extracted features. In order to achieve a good motion classification performance, three popular supervised classification approaches are employed in our research work for the validation and these three classifiers are described as follows.

#### 4.4.1. Decision Tree

**Figure 8:**
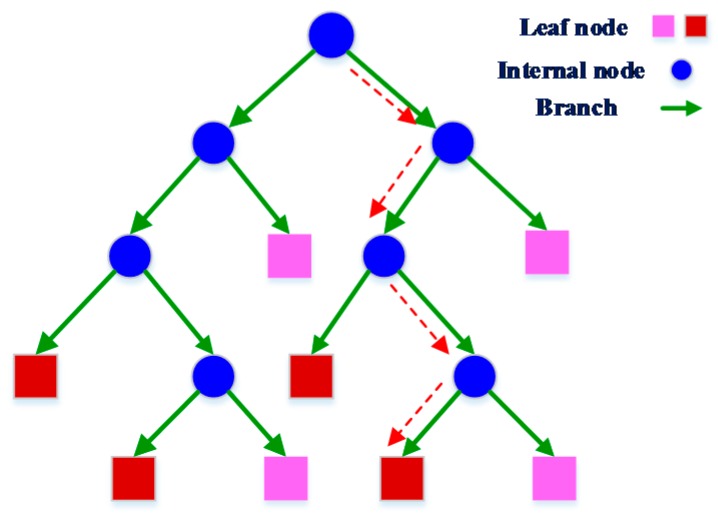
Decision tree graphical model.

A decision tree is a decision support tool that uses a tree-like graph or model of decisions and their possible consequences. Generally, internal node, branch, and leaf nodes are included in a decision tree classifier, where, the internal node represents a test on the selected feature, branch denotes the outcome of the test, and the leaf nodes represent the class labels (different moving directions). Figure 8 graphically illustrates the decision tree model.

Figure 8 draws the graphical model of the decision tree. The blue circles denote the internal node that executes the test on the feature (comparison of the feature with trained parameter), the green arrows denote the test outcomes and the rectangles denote the different labels or classes. The red dashed lines from top nodes to the leaf nodes represent a decision process or classification rule. 

The tree generation is the training stage of this classifier and it works in a recursive procedure. The general tree generation process is that, for each feature of the samples, a metric (the splitting measure) is computed from splitting on that feature. Then, the feature that generates the optimal index (highest or lowest) is selected and a decision node is created to split the data based on that feature. The recursion procedure stops when the samples in a node belong to the same class (majority), or when there are no remaining features on which to split. Depending on different splitting measures, the decision tree can be categorized as: ID3 (Iterative Dichotomiser 3), Quest (Quick, Unbiased, Efficient, Statistical Tree), CART (Classification And Regression Tree), C4.5, etc. [45,46]. 

#### 4.4.2. K-Nearest Neighbors

**Figure 9:**
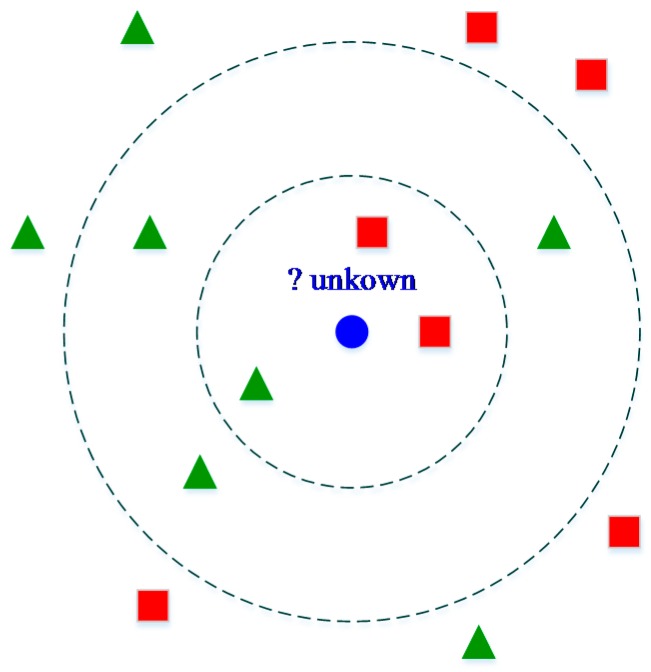
K-nearest neighbors algorithm (kNN) algorithm concept.

K-nearest neighbors algorithm (kNN) [47] is an approach based on the closest training samples in the feature space, where k denotes the number of classes. In the kNN approach, an object is classified by a majority vote of its neighbors, with the object being assigned to the most common class among its nearest neighbors. Similarity measures are fundamental components in this algorithm and different distance measures can be used to find the distance between data points. Figure 9 illustrates the main concept of kNN algorithm.

As shown in Figure 9, the test sample (blue circle) is classified by the neighbors class, either green square or green triangle. If k is selected as 3, the test sample is assigned to the red square because two of its k neighbors belong to red square. In the same way, if k = 5, the test sample is assigned to green triangle class. Hence, the main idea of kNN is that the category of the predicted object is decided by the labels of the neighbors’ majority. Additionally, the votes of these neighbors could be weighted based on the distance to overcome the problem of non-uniform densities of the neighbor classes. 

#### 4.4.3. Support Vector Machine

**Figure 10:**
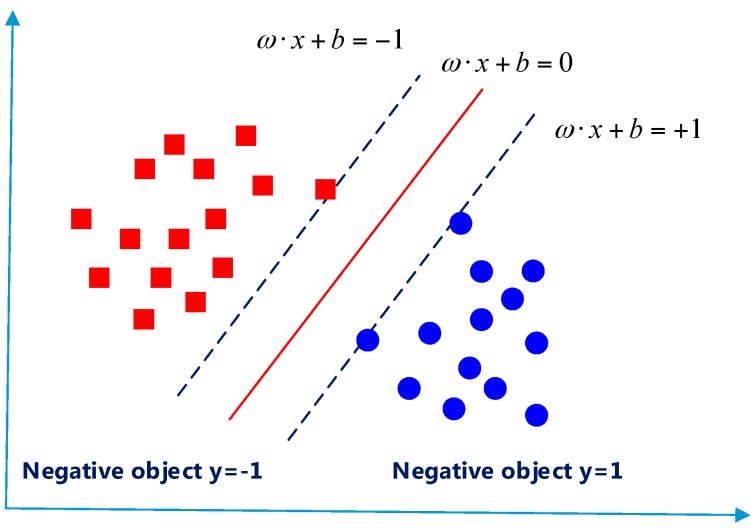
Support vector machine (SVM) classifier.

The support vector machine (SVM) is used to construct a hyperplane or set of hyperplanes in a high- or infinite-dimensional space for classification, regression, or other tasks. Since several available hyperplanes are able to classify the data, the SVM is employed to use the one that represents the largest separation, or margin, between the two classes to classify. The hyperplane chosen in SVM maximize the distance between the plane and the nearest data point on each side. Figure 10 draws the SVM classifier. 

As shown in this figure, the optimal separating hyper-plane (solid red line) locates the samples with different labels (blue circles 1, red square −1) in the two sides of the plane, and the distances of the closest samples to the hyper-plane in each side become maxima. These samples are called support vectors and the distance is optimal margin. The specific illustration of the SVM classifier can be found in the literature [48,49,50].

## 5. Experiments and Results

The experiment is designed to include two parts. In the first part, different testers are invited to perform the five foot motions in their own manners. Then, we collect the data, preprocess data to remove error, divide the data into segments, extract the features and put them into the training process of introduced machine learning algorithms to derive the classifiers. Additionally, the classifiers are tested by two cross validation approaches. In the second part, the data processing procedure is transformed in our software platform and is programmed in C++, the program is also connected to the game control interface to perform the practical game playing experiment.

### 5.1. Date Set

**Figure 11:**
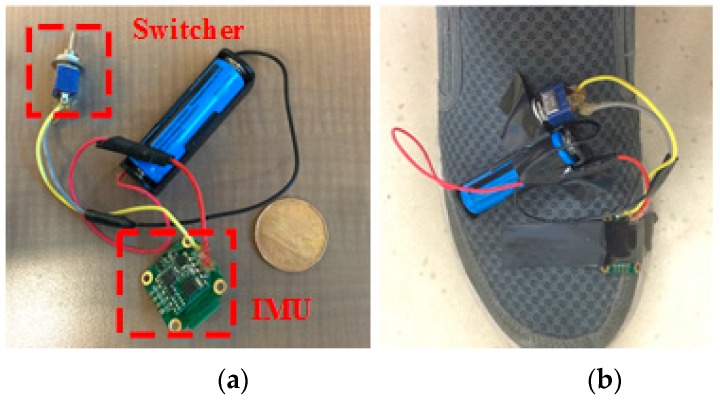

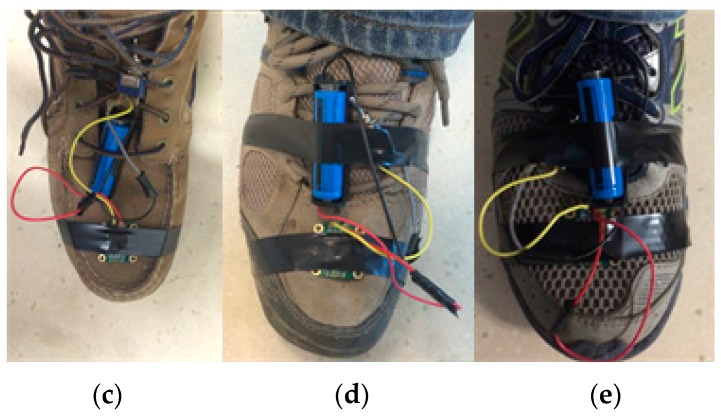
Hardware platform and the sensor placement on shoe. (**a**) Hardware platform of proposed system; (**b**–**e**) Sensor placement on different testers’ shoes.

In order to obtain a sufficient amount of data for training, ten testers—two females and eight males—are invited to participate in experiments. All testers are in good health condition without any abnormality in their gait cycles. The IMU sensor was attached on the testers’ shoes, and they were guided to perform the five stepping motions in their natural manners. In order to have diverse characteristic of each motion, some actions of the testers were conducted at different strengths (heavy or slight), different frequencies (fast or slow), and different scopes (large or small amplitude), and some actions were performed by the same tester on different days. The data collected during this experiment were stored to form the training dataset. Figure 11 shows the system hardware platform. In this platform, a 3.7 V lithium battery (blue one) is used to provide the power supply. The IMU module has a small size, and is very convenient to mount on user’s shoe. Table 1 provides a summary of the collected training dataset, where the quantitative information of the collected human stepping motions is listed in this table. The second row lists the actual motion numbers collected in the experiment, and they include 895 jump, 954 stepping left, 901 stepping right, 510 moving forward and 515 moving backward.

### 5.2. Classification Results

In our proposed system, a corresponding classifier is trained for each motion instead of using a single classifier for the five motions. This training strategy is beneficial to improve the robustness and decrease the complexity of this system, since one classifier only needs to recognize two classes instead of five. Moreover, it offers the possibility of selecting typical features for each motion based on motion principle or data analysis in future work. 

In order to have a better evaluation of the classification performance, two cross-validation approaches for test were chosen: k-fold cross validation and holdout validation. In k-fold cross-validation approach, the original sample is randomly partitioned into k equal sized subsamples. A single subsample is then retained from these k subsamples as the validation data for testing the model, and the remaining (k − 1) subsamples are used as training data. The cross-validation process is then repeated k − 1 times, with each of the k − 1 subsamples used exactly once as the validation data. The k − 1 results from these folds can then be averaged to produce a single estimation. The advantage of this method over repeated random sub-sampling is that all of the observations are used for both training and validation, and each observation is used for validation exactly once. Here, a commonly used 10-fold test is employed. In holdout validation, a subset of observations is chosen randomly from the initial samples to form a validation or testing set, and the remaining observations are retained as the training data. Twenty-five percent of the initial samples are chosen for test and validation. The two cross validation approaches are also performed for the three classifiers and the classification results are listed in Table 2 and Table 3.

For each motion, the column tagged with Class 1 shows the correct detection result of actual motions and the column tagged with Class 0 denotes the undesired jump motion detected from other motions. Specifically, for the jump motion detected by decision tree classifier, 813 motions are successfully identified out of 895 jump motions (where 82 actual jump motions are missed or falsely detected), and 75 motions of totally 2880 other motions (the sum of left, right, forward and backward) are falsely considered as jump motions. 

Additionally, in order to have a quantitative evaluation of the classifier performance, the Accuracy, Precision, and Recall metrics are also introduced. The definition of these metrics and their calculation equations are described below.

- **Accuracy:** The accuracy is the most standard metric to summarize the overall classification performance for all classes and it is defined as follows:
  (12) $$Accuracy=\frac{TP+TN}{TP+TN+FP+FN}$$
- **Precision:** Often referred to as positive predictive value, it is the ratio of correctly classified positive instances to the total number of instances classified as positive:
  (13) $$Precision=\frac{TP}{TP+FP}$$
- **Recall:** Also called true positive rate, it is the ratio of correctly classified positive instances to the total number of positive instances:
  (14) $$Recall=\frac{TP}{TP+FN}$$
  where TP (True Positive) indicates the number of true positive or correctly classified results, TN (True Negatives) is the number of negative instances that were classified as negative, FP (False Positives) is the number of negative instances that were classified as positive and FN (False Negatives) is the number of positive instances that were classified as negative. According to the evaluation metrics, the accuracy, precision, recall, for the test result of each motion are calculated and listed in Table 4, Table 5 and Table 6.

**Figure 12:**
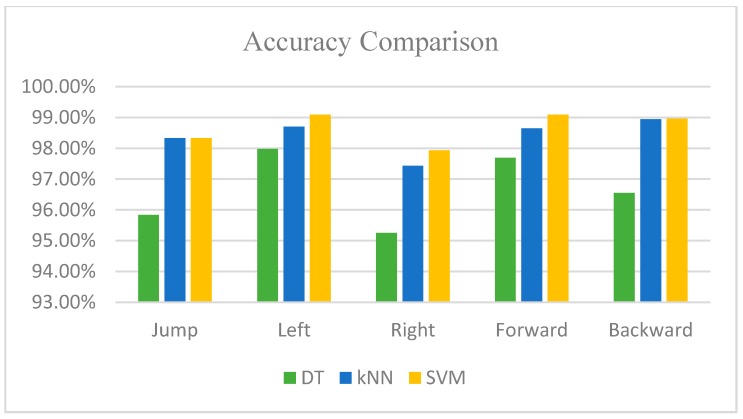
Accuracy comparison of three classifiers.

**Figure 13:**
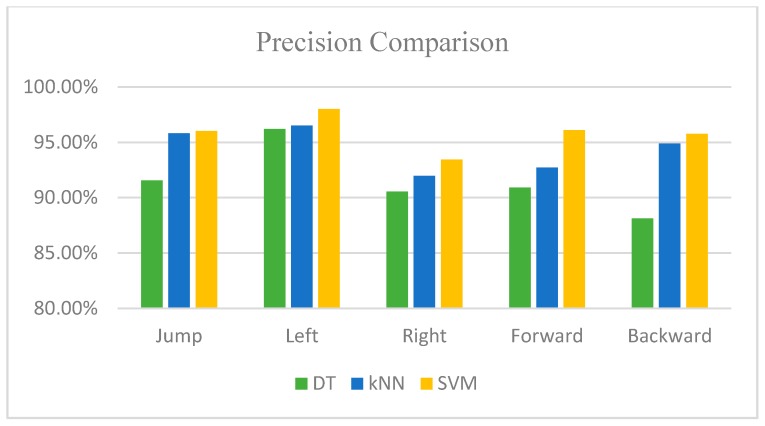
Precision comparison of three classifiers.

Based on the evaluation metrics listed in Table 4, Table 5 and Table 6, and according to the graphically comparison of accuracy and precision shown in Figure 12 and Figure 13, the SVM classifier has an overall better performance than the other approaches. Moreover, the average time for each classifier to make the decision on the motion type is: decision tree classifier 0.0056 ms; kNN, 0.53 ms; and SVM, 0.0632 ms. Although the decision tree classifier has the least response time for identification, its performance on the motion type is not satisfied. The response time for SVM is 0.06 ms and it is in an acceptable time frame because this lag level will not cause an observable delay on user experience. Hence, combined with the performance and the decision time of each classifier, the SVM classifier achieves the best result and is selected in our proposed system to classify the stepping motions. Additionally, we analyze the misclassified events of each motion to give the profile of errors, aiming to avoid that one specific stepping motion always contributes to the wrong recognition, which is potentially due to unsuitable feature selection or data segmentation. The statistical result is listed in Table 7.

Table 7 provides the false identification of each motion in the two cross-validation approaches. For example, in 10-fold cross validation, 27 true jump motions are missing or mistakenly classified, which occupies 42.86% of the misclassified events; however, eight left, seven right, nine forward, and 12 backward are wrongly treaded as jump motion by classifier, which totally contributes 57.15% of the misclassified events. In each classifier, the identification error of its corresponding motion type (i.e., the wrong categorization of jump motion in the jump classifier) occupies approximately 33% to 48%, and the misclassified percentage of other motion varies from 51% to 66%. Moreover, the error result also shows that the misclassified events are averagely distributed in each motion, and demonstrates that no one specific motion error is predominant during the motion determination process. 

### 5.3. Practical Experiment Result

A running game we programmed in Unity is used to practically test the algorithm. In this game, a man is running in the forest with numerous obstacles and the traditional play manner is that the user needs to control the object to jump, go left, go right or get down to avoid the obstacles. Here, we use foot movement direction to control the man and the result is shown in following figures.

**Figure 14:**
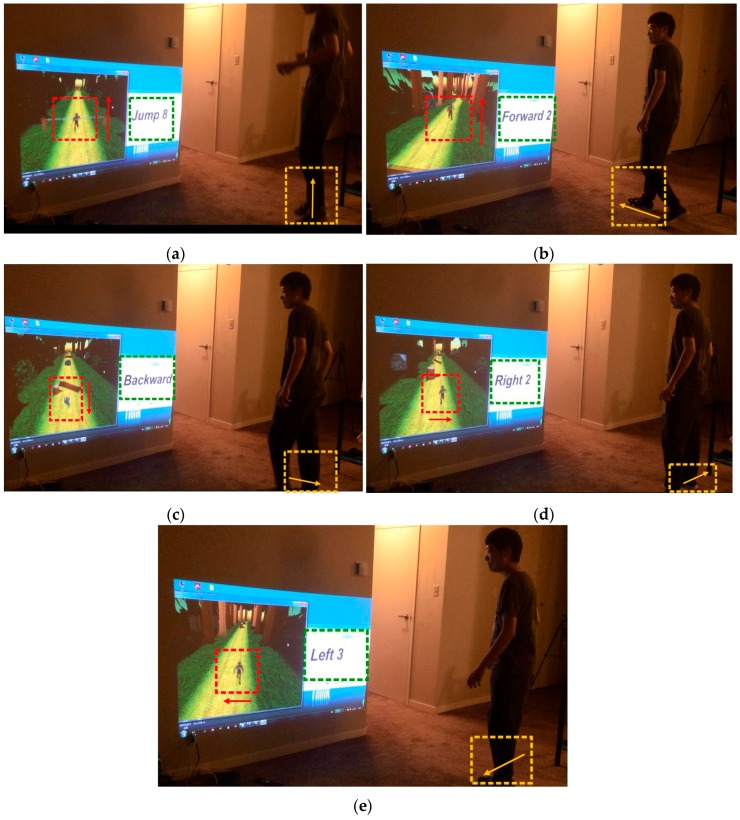
Practical game play test in running game: (**a**) Jump motion; (**b**) Forward motion; (**c**) Backward motion; (**d**) Right motion; and (**e**) Left motion.

As shown in Figure 14, the red rectangle shows the virtual player presented in game, the arrow denotes the player’s moving direction, the green rectangle illustrates the step motion identification result, and the orange rectangle shows the person moving direction. 

**Figure 15:**
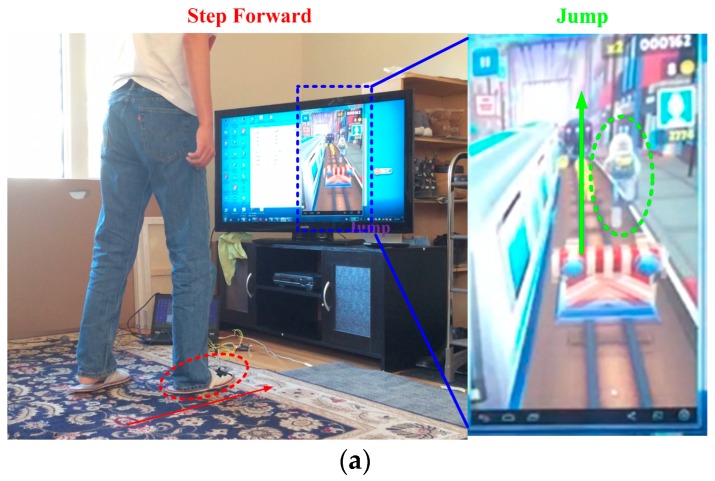

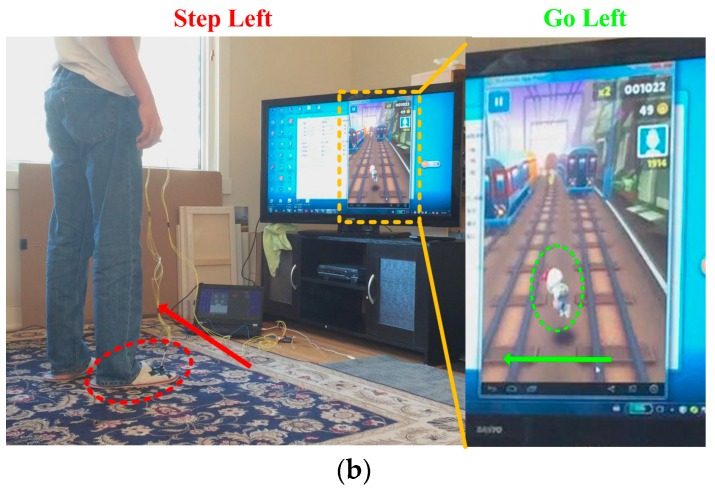
Practical game play test in Game Subway Surfers: (**a**) Step forward; and (**b**) Step left.

Figure 15 shows practical test result in the game Subway Surfers. Figure 15a illustrates that a person walking forward correlates to the jump of kid in game operation. In the left side of this figure, the person steps forward and the red arrow presents the stepping direction. The right side shows the game environment, where we can see that the kid selected in the green circle jumps up to avoid the front obstacle. In the same way, Figure 15b shows the person stepping left and it correlates to the kid moving to left.

## 6. Conclusions

This paper introduces a novel application of foot-mounted inertial sensor based wearable electronic devices—game play. The main contributions of this paper can be summarized as: (1) This paper presents the first attempt to employ user’s stepping direction for controlling the player operation in game play. (2) This paper proposes and implements a novel computationally-efficient, real-time algorithm for the identification of foot moving direction. (3) In the proposed system, the acceleration and gyroscope measurements are fused to derive the attitude and use it to correct the misalignment error. This makes the proposed algorithm compatible with various shoe styles and sensor placements. (4) The stepping motion type can be recognized in the beginning phase of one step cycle, which guarantees the system real-time applicability. (5) It is suggested to design the corresponding classifier for each motion where each classifier only needs to identify two classes instead of using one classifier to recognize all five motions. This is beneficial to acquire a more precise and reliable identification result. (6) Three commonly-used classifiers in the aspects of cross validation performance and response time are compared. Based on this comparison, it is concluded that the SVM classifier achieves the best performance. (7) It extends the inertial sensor based game play scenario to the foot motion control mode, which introduces the possibility of playing running game indoor or anywhere and is potentially beneficial to encourage the user to exercise more for good health. Practical experiments of different users illustrate that the proposed system reaches a high accuracy classification result and excellent user experience, and it effectively broadens the application of current available wearable electronic devices.

## Figures and Tables

**Table 1:** Stepping motion training set.

	Jump	Left	Right	Forward	Backward
Action numbers	895	954	901	510	515

**Table 2:** Classification result of 10-fold cross validation.

	Decision Tree		kNN		SVM	
	Class 1	Class 0	Class 1	Class 0	Class 1	Class 0
Jump	813/895	75/2880	870/895	38/2880	868/895	36/2880
Left	914/954	36/2821	939/954	34/2821	936/954	19/2821
Right	806/901	84/2874	881/901	77/2874	885/901	62/2874
Forward	470/510	47/3265	498/510	39/3265	493/510	20/3265
Backward	445/515	60/3260	502/515	27/3260	498/515	22/ 3260

**Table 3:** Classification result of 25% held out validation.

	Decision Tree		kNN		SVM	
	Class 1	Class 0	Class 1	Class 0	Class 1	Class 0
Jump	212/223	15/718	219/223	8/718	216/223	8/718
Left	226/238	6/703	234/238	15/703	233/238	10/703
Right	198/225	18/716	219/225	16/716	217/225	13/716
Forward	16/127	9/814	125/127	13/814	123/127	7/814
Backward	114/128	7/813	126/128	4/813	126/128	4/813

**Table 4:** Evaluation of Decision tree classifier.

	10-Fold Cross Validation			25% Hold Out Validation		
	Accuracy	Precision	Recall	Accuracy	Precision	Recall
Jump	95.84%	91.55%	90.83%	97.24%	93.41%	95.08%
Left	97.98%	96.21%	95.80%	98.09%	97.41%	94.96%
Right	95.25%	90.56%	89.45%	95.23%	91.67%	88.01%
Forward	97.69%	90.90%	92.15%	97.35%	92.53%	87.45%
Backward	96.55%	88.11%	86.40%	97.77%	94.25%	89.12%

**Table 5:** Evaluation of kNN classifier.

	10-Fold Cross Validation			25% Hold Out Validation		
	Accuracy	Precision	Recall	Accuracy	Precision	Recall
Jump	98.33%	95.81%	97.20%	98.72 %	96.47%	98.20%
Left	98.70%	96.50%	98.42%	97.98%	93.97%	98.31%
Right	97.43%	91.96%	97.78%	97.66%	93.19%	97.33%
Forward	98.64%	92.73%	97.64%	98.40%	90.57%	98.42%
Backward	98.94%	94.89%	97.47%	99.36%	96.92%	98.43%

**Table 6:** Evaluation of SVM classifier.

	10-Fold Cross Validation			25% Hold Out Validation		
	Accuracy	Precision	Recall	Accuracy	Precision	Recall
Jump	98.33 %	96.01%	96.98 %	98.40%	96.42%	96.86%
Left	99.09%	98.01%	98.11%	98.40%	95.88%	97.89%
Right	97.93%	93.45%	98.22%	97.76%	94.34%	96.44%
Forward	99.09%	96.10%	96.66%	98.83%	94.61%	96.85%
Backward	98.96%	95.76%	96.69%	99.36%	96.92%	98.43%

**Table 7:** Foot motion identification error of SVM.

		Jump	Left	Right	Forward	Backward	Corresponding Motion Error	Other Motions Error
10-fold cross validation	Jump	27	8	7	9	12	42.86%	57.14%
	Left	4	18	5	7	3	48.65%	51.35%
	Right	13	15	16	16	18	20.51%	79.49%
	Forward	6	5	4	17	5	45.95%	54.05%
	Backward	7	6	5	2	17	43.59%	56.41%
25 hold out validation	Jump	7	1	4	2	1	46.67%	53.33%
	Left	3	5	2	1	4	33.33%	66.67%
	Right	2	5	8	4	2	38.10%	61.90%
	Forward	1	3	2	4	1	36.36%	63.64%
	Backward	2	1	0	1	2	33.33%	66.67%
